# Analysis of PMMA versus CaP titanium-enhanced implants for cranioplasty after decompressive craniectomy: a retrospective observational cohort study

**DOI:** 10.1007/s10143-022-01874-5

**Published:** 2022-10-12

**Authors:** Dominik Wesp, Harald Krenzlin, Dragan Jankovic, Malte Ottenhausen, Max Jägersberg, Florian Ringel, Naureen Keric

**Affiliations:** Department of Neurosurgery, University Medical Center Mainz, Langenbeckstr. 1, 55131 Mainz, Germany

**Keywords:** Cranioplasty, PMMA, Bioactive titanium-enhanced CaP, Osseointegration, Decompressive craniectomy

## Abstract

Numerous materials of implants used for cranioplasty after decompressive craniectomy (DC) have been investigated to meet certain demanded key features, such as stability, applicability, and biocompatibility. We aimed to evaluate the feasibility and safety of biocompatible calcium-phosphate (CaP) implants for cranioplasty compared to polymethylmethacrylate (PMMA) implants. In this retrospective observational cohort study, the medical records of all patients who underwent cranioplasty between January 1st, 2015, and January 1st, 2022, were reviewed. Demographic, clinical, and diagnostic data were collected. Eighty-two consecutive patients with a mean age of 52 years (range 22–72 years) who received either a PMMA (43/82; 52.4%) or CaP (39/82; 47.6%) cranial implant after DC were included in the study. Indications for DC were equally distributed in both groups. Time from DC to cranioplasty was 143.8 ± 17.5 days (PMMA) versus 98.5 ± 10.4 days (CaP). The mean follow-up period was 34.9 ± 27.1 months. Postoperative complications occurred in 13 patients with PMMA and 6 in those with CaP implants (13/43 [30.2%] vs. 6/39 [15.4%]; *p* = 0.115). Revision surgery with implant removal was necessary for 9 PMMA patients and in 1 with a CaP implant (9/43 [20.9%] vs. 1/39 [2.6%]; *p* = 0.0336); 6 PMMA implants were removed due to surgical site infection (SSI) (PMMA 6/43 [14%] vs. CaP 0/39 [0%]; *p* = 0.012). In this study, a biocompatible CaP implant seems to be superior to a PMMA implant in terms of SSI and postoperative complications. The absence of SSI supports the idea of the biocompatible implant material with its ability for osseointegration.

**Supplementary Information:**

The online version contains supplementary material available at 10.1007/s10143-022-01874-5.

## Introduction

### Background and rational

Cranioplasty after decompressive craniectomy (DC) is a neurosurgical procedure to repair resulting skull defects. Defect reconstruction was historically considered for cosmetic reasons [31] More recent studies suggest a beneficial role for the patient’s functional and neurological outcome [34, 37]. Both motor and cognitive functions improve after cranioplasty due to cerebrospinal fluid hydrodynamics and cerebral blood flow changes [12, 17, 31]. Although the surgical procedure is relatively straightforward, it is associated with substantial cost and morbidity [44]. Complications include post-operative bleeding, seizures, meningitis, surgical site infection (SSI), and bone flap resorption (BFR) [6, 10, 38, 42]. Uncertainty persists regarding the implications of the timing of cranioplasty in terms of complication rates and potential benefits [2, 3, 40, 45]. Materials for reconstruction of cranial defect ideally provide osteoinductive and osteoconductive properties to promote structural and functional restoration [32]. Autologous bone flap reinsertion is common after hemicraniectomy. Storage of the bone flap between craniectomy and cranioplasty is performed either intracorporeally (e.g., the patient’s abdomen) or extracorporeally (i.e., by tissue banking) [27]. Furthermore alternative storage methods of extracorporeal preservation of the bone after sterilization in an autoclave or in ethylene oxide at room temperature have also been reported [28, 29]. Alloplastic materials, such as polymethylmethacrylate (PMMA), polyetheretherketone (PEEK), polyethylene, titanium, and injectable/moldable calcium phosphate-based bone cement have been used as alternatives [4, 39, 41, 47]. Risk factors for SSI, implant exposure, and graft removal after alloplastic cranioplasty include inaccurate matching and poor bone and soft tissue integration [1, 30]. In a large meta-analysis comparing different allografts, PEEK offers a low infection rate (5%; 95% CI 0–11) in 5 studies and is superior to autografts (RR 0.20; 95% CI 0.07–0.57), hPMMA (RR 0.20; 95% CI 0.07–0.60), Ti (RR 0.39; 95% CI 0.17–0.92), and pPMMA (RR 0.14; 95% CI 0.04–0.51) [19]. Infection occurred in 8% (95% CI 6–11) and implant exposure in 6% (95% CI 4–9) [19]. The stringency of data on the safety and complication rates of various materials used in cranioplasty is limited by a large diversity of study methods, clinical settings, and reported outcomes [41]. Nonetheless, studies agree that the failure risk of autografts is higher than that of allografts [18, 19, 26, 41].

With the developments of 3D virtual planning and computer-aided design and manufacturing based on individually computed tomography (CT), precisely fitted patient-specific alloplastic implants are now available for reconstruction of the bony defect [32, 39]. By incorporating innovative osseointegrative, osteoconductive, and osteoinductive biomaterials, these custom implants may alleviate the shortcomings mentioned above. A novel bioactive calcium phosphate (CaP) titanium-enhanced cranial implant by OssDsign (Uppsala, Sweden) has been shown to induce bone healing demonstrated by gene expression analyses and histology in patients with cranial defects [13]. These custom bioactive implants show potential for overcoming current issues with alloplastic implants, leading to improved patient care and outcomes.

### Objective

It is hypothesized that bioactive implants leads to improved implant incorporation and fewer surgical site infections, thus providing improved outcomes. Our study evaluates the feasibility and safety of biocompatible CaP titanium-enhanced implants for cranioplasty compared to alloplastic PMMA implants in cranioplasty.

## Methods

According to the local laws of Rhineland Palatinate, Germany (Landeskrankenhausgesetz §37), no formal approval and informed consent is necessary for such kind of retrospective analysis. Patients consented prior to the procedure.

### Study design and patient population

All patients who received either a PMMA or a CaP cranial implant between January 1st, 2015, and January 1st, 2022, were included in this retrospective single center observational study. As standard of care (SOC) patients subjected to cranioplasty before 2019 received PMMA implants and CaP implants from January 2020 onward. All autologous bone flaps were discarded after hemicraniectomy due to local hygiene regulations, so that the cranioplasty procedure with the PMMA or CaP implants was primary and not rescue surgeries. The standard surgical procedure remained unchanged when switching the implant material. (Supplement 1) Demographic, clinical, and diagnostic data were collected before and after the cranioplasty. Early (< 72 h) postoperative CT scans were used to evaluate the implant fit and occurrence of complications. Hemorrhage, CSF fistulas, seizures, implant loosening and dislocation, wound healing disorders, and SSI were defined as post-operative complications. Compromised implant fitment and failure of implant integrity were defined as intraoperative complications. Long-term complications included hemorrhage, SSI, wound dehiscence, and implant dislocation. SSI infections are defined as any infection occurring after surgery in those areas where the procedure took place. In case of explanation, integration was assessed by the individual surgeon according to 3 main characteristics: (1) presence of a clear cleavage plain, (2) degree of vascularization, and (3) difficulty to separate and removal of the implant.

### Follow-up

Early (< 72 h) postoperative CT scans were performed in each patient. Patients were either re-evaluated in our outpatient department, or their relatives were contacted by phone to obtain an assessment of the evolution of the neurological status and potential complications after cranioplasty as a part of the clinical routine.

### Bias

To account for difference in follow-up time, we performed a subgroup analysis of 20 patients from each group with a given 1-year follow-up in our outpatient department. The manuscript has been prepared according to the “STROBE” checklist for observational studies as far as methodology allows (Supplement 2).

### Statistical methods

Data analysis was performed using GraphPad Prism version 8.4.2 for macOS, GraphPad Software, La Jolla, CA, USA, www.graphpad.com. Unpaired categorical and binary variables were analyzed in contingency tables using Chi-square and Fisher’s exact test. The log-rank test was performed to assess differences between both study groups (survival curves). Cox proportional hazards regression was used for multivariate analysis. Findings were reported as mean or mean ± SD/SEM. Results with *p* < 0.05 were considered statistically significant.

### Implants for cranioplasty

**Fig. 1:**
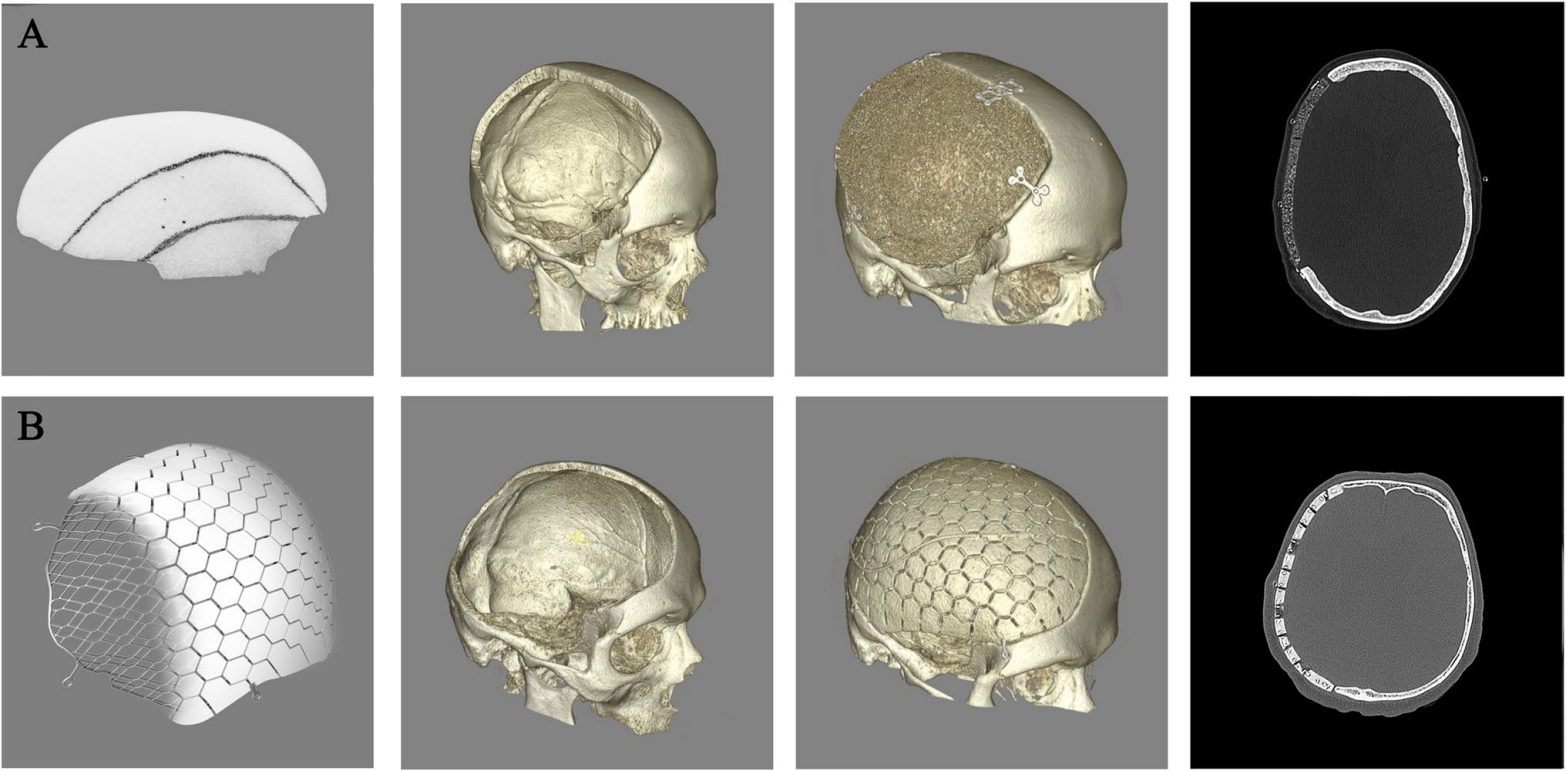
PMMA implant (**A**) and bioactive CaP implant (**B**). Ceramic implants provided a superior matching of the craniectomy defect

All implants were designed from high-resolution (1.0-mm-thick slices) CT scans and returned to the surgeon for approval before production. Mosaic-designed CaP titanium-enhanced were manufactured using a molding technique as described previously (OssDsign, Uppsala, Sweden) [14]. They are constructed by an inner titanium mesh to enhance stability and coated by a biocompatible CaP shell. A porous fine-grained PMMA material characterizes the PMMA implants (Zimmer Biomet, Warsaw, USA). The porous structure has been designed to allow fibrovascular ingrowth and bony attachment, while its rigidity equals the skull bone [33]. PMMA implants were likewise custom made from high-resolution (1.0-mm-thick slices) CT scans (Fig. 1).

## Results

### Participants and baseline characteristics

We included 82 consecutive patients who received either a PMMA or CaP cranial implant after decompressive craniectomy (DC). Forty-three consecutive patients received a PMMA implant between January 1st, 2015, and December 31st, 2018. Further, 39 consecutive patients received a CaP cranial implant between January 1st, 2020, and January 1st, 2022. DC was performed mainly due to malignant middle cerebral artery infarction (PMMA: 20/43 [46.5%] vs. CaP: 9/39 [23.1%]), traumatic brain injury (PMMA: 15/43 [34.9%] vs. CaP: 10/39 [25.6%]), subarachnoid hemorrhage (PMMA: 5/43 [11.6%] vs. CaP: 15/39 [38.5%]), or intracerebral hemorrhage (PMMA: 2/43 [4.7%] vs. CaP: 5/39 [12.8%]). There was no significant difference in sex (PMMA: 14/43 [32.6%] females and 29/43 [67.4%] males vs. CaP: 20/39 [51.3%] females and 19/39 [48.7%] males; *p* = 0.067) and mean age (PMMA: 51.2 ± 11.2 years; CaP: 53 ± 12.4 years; *p* = 0.81) in both groups. Similar numbers of patients received DC of either the left or right hemisphere. Although it was not significant, the time from DC to cranioplasty was different in the two groups (PMMA: 143.8 ± 17.5 days vs. CaP: 98.5 ± 10.4 days; *p* = 0.102) (Table 1). Patients requiring persistent ventricular drainage due to hydrocephalus occurred equally in both groups (PMMA:14/43 [32.6%] vs. CaP: 14/39 [35.9%]; *p* = 0.818). Patients with a ventricular-peritoneal shunt were not prone to higher numbers of infection or surgical complications.

**Table 1:** Baseline demographics and patient characteristics

	PMMA	CaP
Study population (No. of patients)	43	39
Mean age (SD)	51.2 (11.2)	53 (12.4)
Sex		
Female	15	22
Male	28	17
Diagnosis (nb of patients (%)		
MCA infarction	20 (47)	9 (23)
SAH	5 (12)	15 (38)
TBI	15 (35)	10 (26)
ICH	2 (5)	5 (13)
Others	1 (2)	
Side of decompression		
Right	25	18
Left	18	21
Time to cranioplasty in days (SEM)	143.8 (17.5)	98.5 (10. 4)
Number of surgeons involved	23	20
Modified Rankin scale (mRS; median; range)	4 (1–4)	4 (1–4)
American Society of Anesthesiologists (ASA) score (median; range)	3 (2–4)	4 (1–4)
Diabetes (no of patients)	2	7
Smoking (no of patients)	6	6
Previous radiation (no of patients)	1	0
Previous cranial surgeries		
1	22	14
2	13	15
> 2	8	10

### Outcome and complications

The mean follow-up period were 49.3 ± 22.8 months for the PMMA and 8.1 ± 4.9 months for the CaP implant group. There were no intraoperative complications related to the implant itself in both groups. Intraoperative matching was excellent with both implant types; however, the CaP implants appeared to be superior as they retain partial modelability. A post-operative scan showed the near perfect fit of the implant without detectable offset or gaps (Fig. 1, supplemental Fig. 2).

**Fig. 2:**
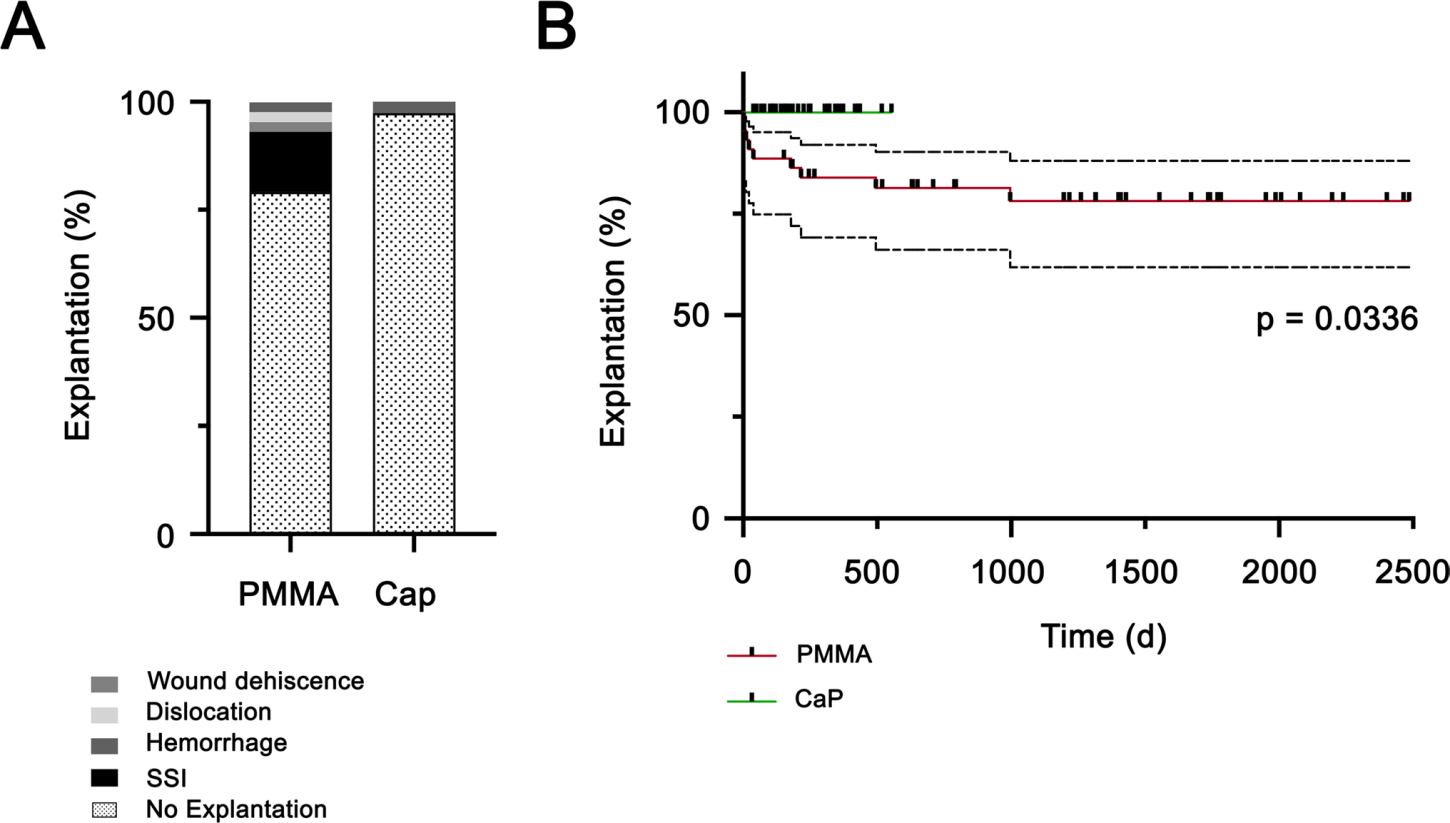
Postoperative complications after cranioplasty Implant explantations during follow-up (**A**). Survival analysis for explantation probability (**B**)

After cranioplasty, 19 postoperative complications occurred in 13 patients with PMMA implants (5 epidural hematoma (EDH), 5 seizures, 6 SSI, 1 insufficient matching implant, 1 CSF fistula, and 1 wound dehiscence) and in patients with CaP implants (4 EDH, 2 with EDH and seizures) (Table 2). SSI occurred in 6 PMMA patients (immediately after surgery in 4 patients, 1 within first year and 1 during the second year after cranioplasty). No surgical site infection was observed after cranioplasty with CaP (PMMA 6/43 [14%] vs. CaP 0/39 [0%]; *p* = 0.012). After PMMA implant cranioplasty, revision surgery with implant removal was necessary for 9 patients (9/43; 20.9%), while implant removal was performed due to SSI (6 patients), wound dehiscence (1 patient), implant dislocation (1 patient), and epidural hematoma (1 patient) (Fig. 2A). One patient (1/39; 2.6%) with a CaP implant necessitated revision surgery with explanation due to skin atrophy developed by pathological head posture. No bacterial growth was detected on the implant itself. There was no statistically significant difference in the occurrence of postoperative complications in both groups (PMMA 13/43 [30.2%] vs. CaP 6/39 [15.4%]; *p* = 0.1). However, the PMMA implants were removed significantly more often than CaP implants (PMMA 9/43 [20.9%] vs. CaP 1/39 [2.6%]; *p* = 0.0336), which was confirmed by the survival analysis (Fig. 2B).

**Table 2:** Postoperative complications

	PMMA	CaP	Total
Postoperative complications (%)			
Epidural hematoma	5 (11.6)	4 (10.3)	9 (11)
Seizures	5 (11.6)	2 (5.1)	7 (8.5)
Insufficient matching	1 (2.3)	0	0
CSF fistula	1 (2.3)	0	1 (1.2)
Early SSI	4 (9.3)	0	4 (4.9)
Long-term complications (%)			
SSI	2 (4.6)	0	2 (2.4)
Wound dehiscence	1 (2.3)	1 (2.6)	2 (2.4)
Total number of complications/patients	19/ 13	6/6	25/19

**Fig. 3:**
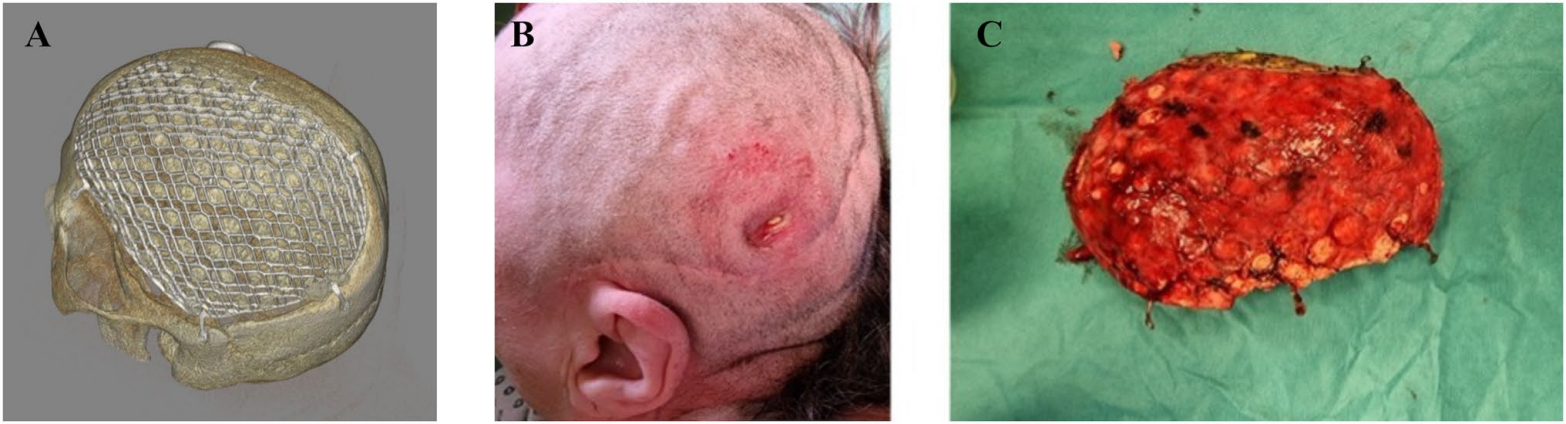
CT scans (**A**) prior to the explantation of a bioactive ceramic implant due to contaminated atrophic skin lesion from pathological head posture leading (**B**). No bacterial infection was detected on the implant itself. The implant showed signs of strong osseointegration with the surrounding tissue (**C**)

After explantation, less integration, as indicated by a clear, non-vascularized cleavage plain enabling easy removal was macroscopically observed in PMMA implants. In contrast, the CaP implant was firmly integrated in a vastly vascularized layer on both sides of surrounding tissue penetrating the implant. Integration in bioactive implants was pronounced with the surrounding tissue rendering the implant hard to separate from the elated skin necessitating sharp dissection. The CaP implants showed an outstanding anatomical matching in the osteoclastic defect in this series due to the possibility of intraoperative partial modeling (Fig. 3). MRI images 6 months after implantation showed good integration and vascularization of the covering skin flap (supplemental Fig. 1).

In a multivariable logistic regression model adjusted for follow-up time, sex, age, pre-operative mRS, and ASA grade, PMMA implants had a higher likelihood of removal (OR 3.12, 95% CI = 2.2–1914, *p* = 0.04).

A proportional hazard model was performed including the mentioned known risk factors such as mRS at admission, age, and ASA classification. Here, implant material (HR = 9.26, 95% CI = 1.09–233.9) and mRS > 3 (HR = 1.29, 95% CI = 0.25–5.26) were predictive of increased implant removal.

### Subgroup analysis

Thirty-five patients that received either a PMMA or CaP implant after DC were included in our subgroup analysis. Twenty consecutive patients between January 1st, 2017, and December 31st, 2017, received a PMMA implant. Further, 15 consecutive patients received a bioactive CaP implant between January 1st, 2020, and December 31st, 2020. DC was performed due to malignant middle cerebral artery infarction (PMMA: 13/20 [65%] vs. CaP: 4/15 [26.7%], subarachnoid hemorrhage (PMMA: 3/20 [15%] vs. CaP: 8/15 [53.3%], intracerebral hemorrhage (PMMA: 2/20 10%] vs. CaP: 1/15 [6.7%]), or traumatic brain injury (PMMA: 2/20 [10%] vs. CaP: 2/15 [13.3%]). During the follow-up of 12 months, 8 postoperative complications occurred in patients with PMMA implants (3 patients with EDH, 3 with seizures, 1 CSF fistula, 1 implant dislocation) and 3 in those with CaP (2 patients with EDH, 1 with seizures). Revision surgery with implant removal was required in 5 patients with PMMA (all due to SSI), none in those with ceramic implants within 1 year after cranioplasty (PMMA 5/20 [25%] vs. CaP 0/15 [0%]; *p* < 0.00365).

## Discussion

### Key findings

The present study provides evidence that implants with osteoconductive and osteoinductive properties can reduce the risk of SSI and the rate of explantations after cranioplasty compared to allogenic materials (PMMA). There was no significant difference in surgery-related complications between the two implant types in our study. Bioactive CaP implantation showed superior integration and vascularization of the covering tissue.

### Limitations

This is a single-center retrospective observational analysis including all consecutive patients subjected to cranioplasty, and its findings must be interpreted in consideration of this with caution. As is the challenge for cranioplasty and bioactive materials in general, the level of evidence is low. Bioactive CaP is a relatively young and novel addition to the materials used for, and little is known about long-term durability and integration. To account for this lack in follow-up time, we performed a subgroup analysis of 20 PMMA and 15 CaP patients with a given 1-year follow-up in our outpatient department. Another limitation is the use of historical controls which is necessary as since 2020 only the described CaP implants have been used for cranioplasty in our institution.

### Interpretation

The optimal timing of cranioplasty to minimize complications has yet to be established. Some favor early (within 12 weeks) and ultra-early (within 4 weeks) cranioplasty [12, 26, 31]. Early cranioplasty has been associated with lower infection rates and lower probability of developing hydrocephalus, although some authors reported that timing had no influence and have observed similar SSI numbers [22, 31]. In our study, cranioplasty was performed at 122.2 ± 96 days after DC. The time span did not differ between both groups. Continued research will show if the timing of cranioplasty should be adapted to the chosen implant material.

Titanium-enhanced biocompatible CaP implants comprise a ceramic compound containing monetite, β-calcium pyrophosphate (PPi), β-tricalcium phosphate, and brushite [14]. These ceramics have chemical resemblance to the osteoconductive and osteoinductive elements in native bone. While osseointegration has been defined as load-bearing integration without loosening, osteoconduction facilitates bone growth on a particular surface [35], and osteoinduction encompasses processes leading to the differentiation of undifferentiated osteoprogenitor cells to osteoblasts [30, 32]. Gene expression analysis in bioactive CaP implants has detected osteoblastic activity and bone formation at 9 months after cranioplasty [13, 14]. In large animal models, bioactive ceramics are better promoters of bone formation, remodeling, and osseointegration than titanium implants [14]. Similar properties such as osteoconductive properties, with a high degree of tissue ingrowth and vascularization, have long been associated with HA implants [5, 19]. We found that the integration of the bioactive ceramic implant as seen in one patient after explantation proved superior to PMMA implants. This observation adds proof to the previously published in vitro and animal data. Only 1 patient required explantation of the bioactive ceramic implant. Osseointegration is also observed with PMMA in animal models [7]. However, it is much less pronounced and matter of ongoing research using different porosities and additives such as strontium containing borate bioactive glass to improve upon this property [7, 11]. As HA and CaP implants share similar properties, it seems plausible that improved integration and lower SSI rates have been observed in a prospective randomized trial [25]. Much is yet to be learned about the safety and efficacy of bioactive CaP, among others, as cranioplasty implant materials. Specific advantages and disadvantages compared to biocompatible or alloplastic materials will have to be determined over time. Despite the early paucity in literature, PEEK cranioplasty seems to be associated with lower post-operative complication rates compared to PMMA, titanium, and autografts [19]. Nevertheless, compared to titanium mesh implants, infection rates are high among patients receiving custom PEEK implants [36]. Previous studies indicate that infection and complication rates in cranioplasty with bone cement are substantially higher, while titanium-based implants impair follow-up imaging [23]. We observed no statistically significant difference in postoperative complications such as hemorrhage, CSF fistulas, and seizures. However, the necessity for explanation of PMMA implants was higher in the entire cohort, as well as in a subgroup analysis with a given 1-year follow-up. Surgical site infection and wound dehiscence were the main reasons for implant removal in the PMMA group (6 out of 43 patients, 13.95%). This observation falls in line with previously published data reporting postoperative infection rates using PMMA implants of 14.4% < 3 months and 28.1% > 3 months, respectively [46]. As there is currently no other published literature comparing bioactive CaP implants with alloplastic materials, it remains to be seen how these implants will ultimately reduce complication and implant failure rates and improve clinical outcomes [33]. Prospective clinical trials in this field are difficult to establish due to a tremendous variability of techniques and applied materials. This is also confirmed by the small number of prospective clinical trials performed to date [20, 21]. However, prospective registries (e.g., German Cranial Reconstruction Registry) bear the potential of longitudinal multicentric analyses with homogenous datasets [16, 38]. First data from the Multicenter Prospective Registry of Cranioplasty in the UK and Ireland offers insights into international variation in practice [15]. Here, the most common material used was titanium (64%) followed by autologous implants (14%). The median time to cranioplasty was higher compared to our cohort with about 244 days [15]. The authors report a 30 days readmission rate of 5.5% with 4% being due to SSI [15]. This is almost identical to the observations made in this study, with 4 out of 10 readmissions occurred within 30 days and all being due to SSI. However, the Registry of Cranioplasty in the UK and Ireland does no offer insight into differences between PMMA and CaP implants. It remains to be seen, if similar rates of re-admission and revision surgery become evident once data on longer follow-up intervals is available.

In contrast to alloplastic implants, surgical dissection of ceramic implants was much more difficult due to substantial integration with the surrounding tissue [13, 14]. Extensive adhesions necessitated sharp dissection from the covering skin flap. This seems to further support the potential for improved implant stability and reduced probability of dislocation. It is also of note that despite contamination of the atrophic skin dehiscence, no bacterial contamination was found on the underlying ceramic implant. Implant-associated infections involve biofilms that are challenging to eradicate [9]. Despite biofilm-active antibiotic therapy, implant removal is necessary in most cases [8]. It is possible that antimicrobial treatment will be more effective in bioactive implants. One reason why medical devices get colonized by bacterial biofilms is that a considerably lower bacterial load is needed in comparison to native tissue [24]. Another explanation is the lack of vascularization, rendering implants more susceptible than other tissues and organs in the human body [24]. In theory, bioactive CaP implants overcome the shortcoming by promoting neovascularization, reducing the occurrence of biofilms, and improving the delivery of drugs such as antibiotics to the site of infection [24, 43]. Strong osseointegrative properties also relate to vascularization and soft tissue coverage, and an excellent soft tissue coverage due to robust osseointegration, promotion of vascularization, and tissue ingrowth via multiple interconnected spaces should facilitate improved wound healing, prevention of atrophy, and, with them reduced risk of SSI [14].

There was no difference in rates of postoperative complications such as epidural hematoma or seizures. This is not unexpected, as the main osseointegrative, osteoconductive, and osteoinductive advantages of the novel bioactive compound come to bear over time without immediate impact on the postoperative course [13, 14].

### Generalizability

As always, caution is a prime requirement when drawing conclusions from results in small patient groups. Further research will show if these promising results can be confirmed in larger and prospective trials. Here, CaP implants reduce the risk of SSI and the rate of explantations after cranioplasty compared to allogenic materials independent from time of cranioplasty and underlying cause for hemicraniectomy. It is expected that differences of standard of care should not vary the presented results. Although implant-associated late infections are known and reported, they were not observed in our patient population with the CaP implants during an adequate follow-up period [16, 26]. More extended follow-up periods should determine whether bioactive CaP implants retain their advantages regarding infection and clarify the treatment strategies. The presented data reflects the current state of knowledge and may thus be of use for clinical decision-making as it stands.

## Conclusion

In our study, bioactive CaP implants showed lower rates of SSI requiring explantation. Vigorous osseointegration may be a key factor in implant durability and improved wound healing capability.

## Supplementary Information

Below is the link to the electronic supplementary material.

Supplementary file1 (DOCX 34 KB)

**Supplemental Figure 1**: T2 weighted contrast enhanced MRI images after CaP cranioplasty depicting good osteointegration and vascularization.

High Resolution Image (TIF 7.29 MB)

**Supplemental Figure 2**: Volumetric gap analysis of 10 patients in each group.

High Resolution Image (TIF 69.7 KB)

## Data Availability

All data are available from the corresponding author on reasonable request.
