# Supplementary figures and images for: Analysis of PMMA versus CaP titanium-enhanced implants for cranioplasty after decompressive craniectomy: a retrospective observational cohort study
Source: Neurosurg Rev. 2022 Oct 12;45(6):3647–55. doi: 10.1007/s10143-022-01874-5 (PMC9663391; doi:10.1007/s10143-022-01874-5)

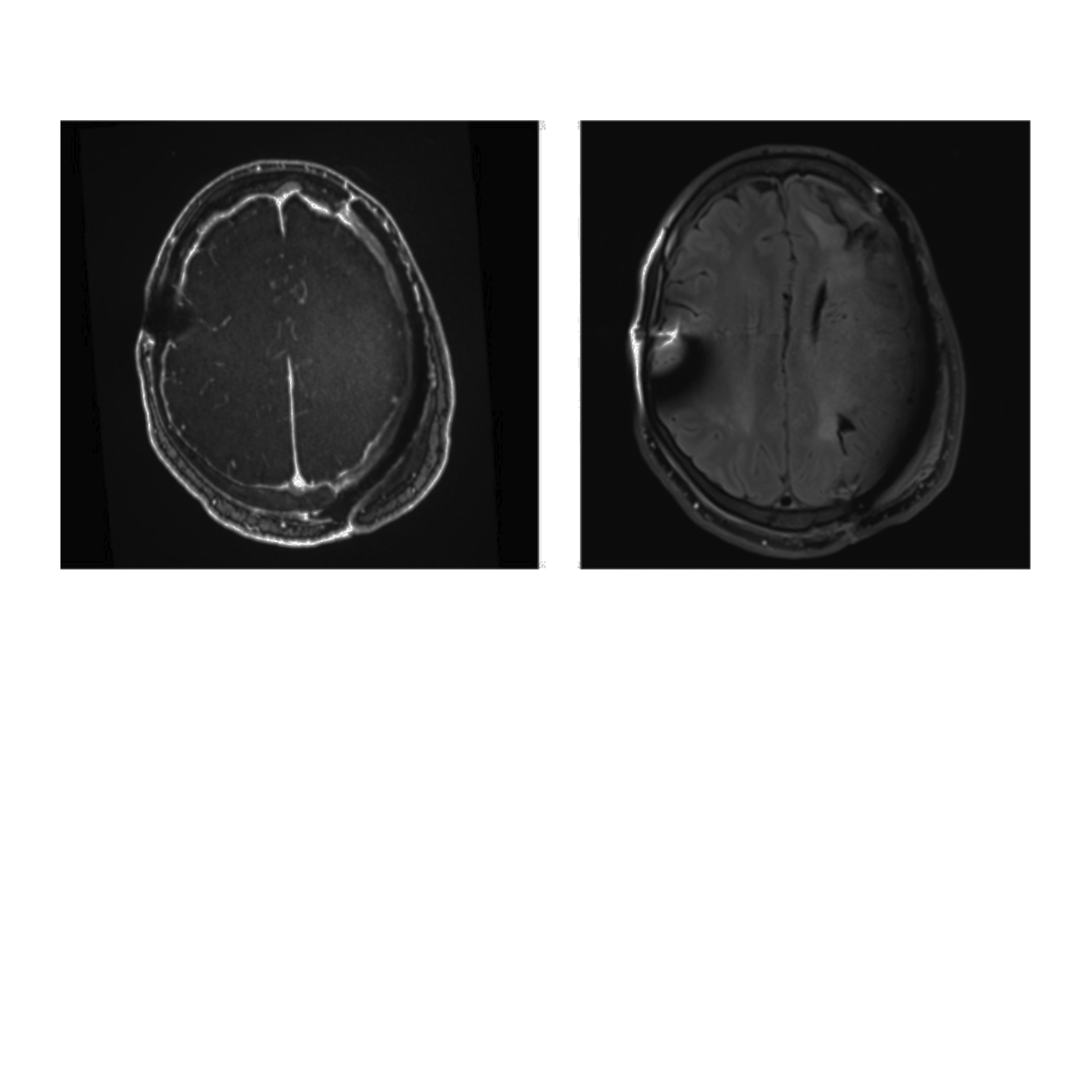

Supplement: Supplementary file 2 — Supplemental Figure 1: T2 weighted contrast enhanced MRI images after CaP cranioplasty depicting good osteointegration and vascularization. [file 10143_2022_1874_Fig4_ESM.png]

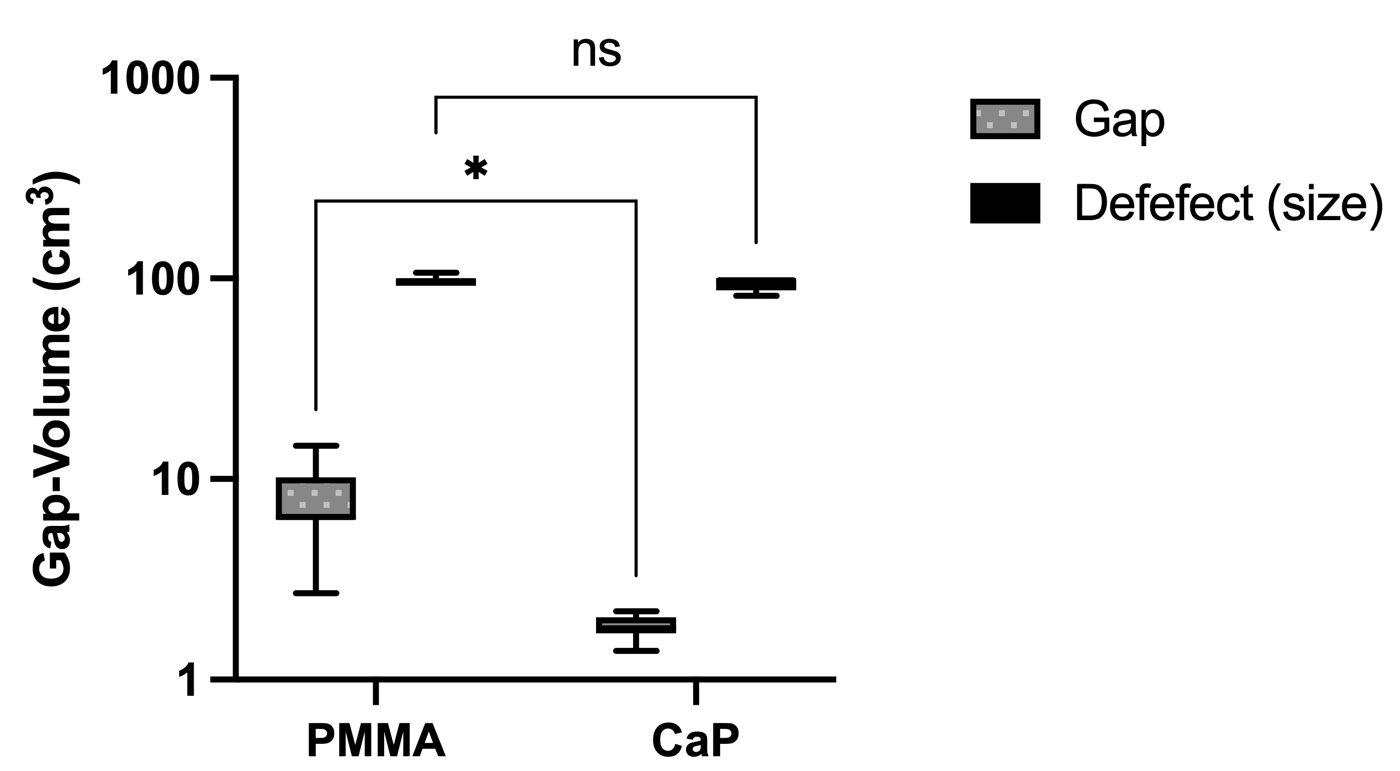

Supplement: Supplementary file 4 — Supplemental Figure 2: Volumetric gap analysis of 10 patients in each group. [file 10143_2022_1874_Fig5_ESM.png]

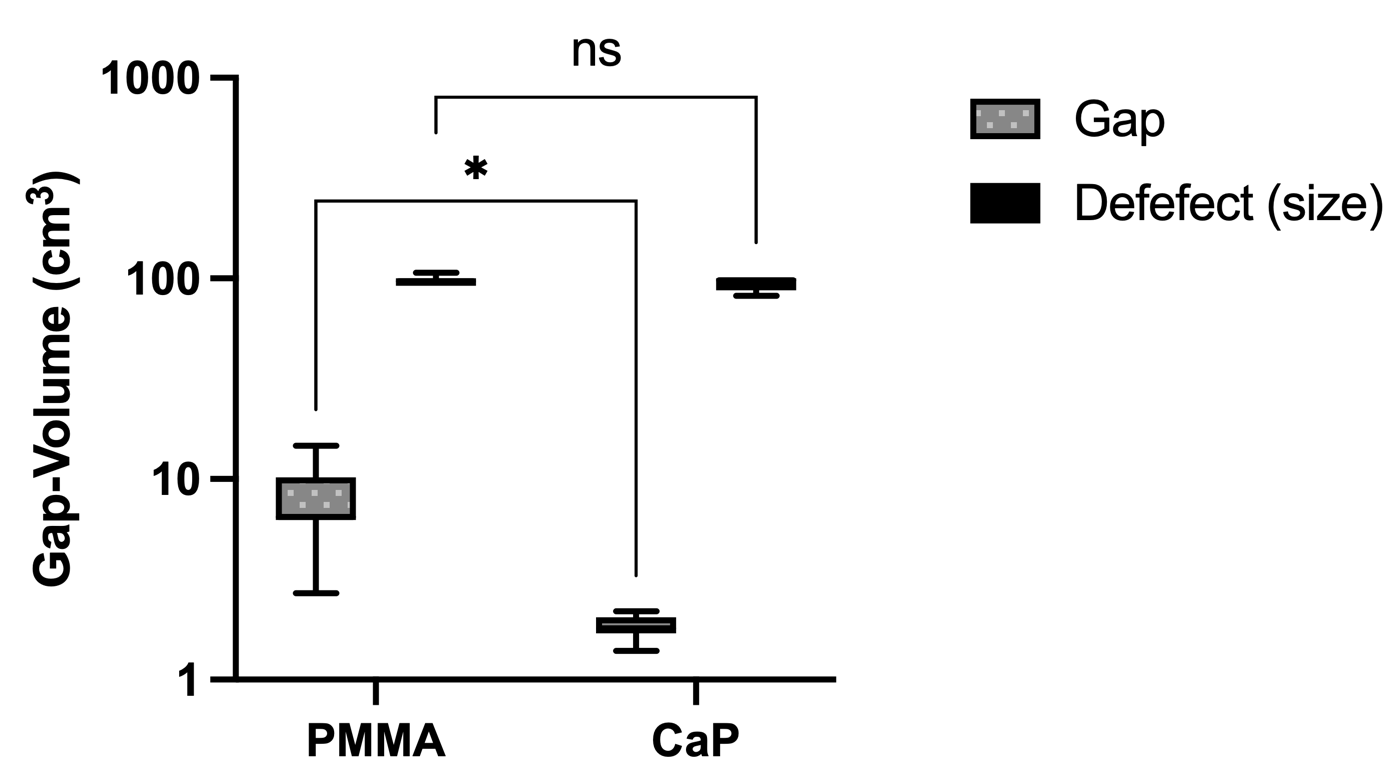

Supplement: Supplementary file 5 — High Resolution Image (TIF 69.7 KB) [file 10143_2022_1874_MOESM3_ESM.tif]
